# Interactive effects of mineral nitrogen rates and silver nanoparticle sprays on ear rot disease and yield of corn in sandy soil

**DOI:** 10.1186/s12870-026-08745-x

**Published:** 2026-04-24

**Authors:** Salah Elsayed Attia Toaima, Sherif Ibrahim Abdel-Wahab, Tamer Ibrahim Abdel-Wahab

**Affiliations:** Crop Intensification Research Department, Field Crops Research Institute, Agricultural Research Center, Postal box 12619, Giza, Egypt

**Keywords:** Mineral N fertilizer, AgNPs, Ear rot disease, Corn yield, Sandy soil

## Abstract

The study draws attention to the potential advantages of silver nanoparticles (AgNPs) in plant nutrition systems through its antibacterial, catalytic and UV-blocking qualities. AgNPs were synthesized by reducing silver ions in a silver N-(2-ethylhexyl) carbamate solution with microwave assistance. The resulting AgNPs of controlled size were characterized using UV-visible spectra, TEM, EDX, and zeta potential analysis. These findings led to the implementation of a field trial at the Ismailia Agricultural Research Station, Agricultural Research Center (ARC), Egypt during the summers of 2022 and 2023 to optimize crop yield while minimizing ear rot disease incidence through the proper mineral nitrogen (N) rate application and AgNPs sprays. Split-plot distributions in a randomized complete blocks design were used in three replications of the experiment. The main plots represented mineral N fertilizer rates (240, 264, and 288 kg N ha^-1^), and the subplots represented AgNPs treatments (the control treatment “without AgNPs spray” and 25, 50, and 75 ppm of AgNPs).The results demonstrated that increasing N rates significantly enhanced corn growth and yield components, with the best rate being 288 kg ha^-1^, leading to reduced ear rot disease incidence and severity. Corn showed the highest yield traits when AgNPs were sprayed at a rate of 75 ppm when corn plants received 288 kg N ha^-1^. Additionally, the incidence and severity of ear rot disease were significantly reduced. The application of 288 kg N ha^-1^ and 75 ppm of AgNPs led to the highest grain yield and the lowest incidence and severity of ear rot disease in corn under sandy soil conditions.

## Introduction

 Crop intensification is categorized into two main approaches horizontal and vertical intensification. Horizontal intensification involves growing multiple crops on a single piece of land (intercropping, crop sequence, and crop rotation). Meanwhile, vertical intensification aims to enhance the productivity of individual crops through techniques such as improved irrigation and fertilization [1]. These approaches are employed to optimize agricultural yield while reducing environmental impact. Corn (*Zea mays* L.) plays a significant role in generating income and driving economic activity in rural areas of Egypt [2]. In 2022, the total cultivated area for yellow corn in Egypt reached approximately 240,000 hectares, with an average yield of 7.50 tons per hectare [3]. However, ear rot is a significant biotic barrier to increasing corn production in Africa, as it can reduce yields by up to 48% [4]. Fusarium species-induced ear rot is a significant concern for corn farmers in Egypt [5].The impact of *Fusarium verticillioides* inoculation on corn ear length, and Fusarium ear rot was assessed across different N fertilization levels [6].

Silver nanoparticles (AgNPs) are extensively applied across various industries as a key product of nanotechnology and can be synthesized through chemical, physical, photochemical, and biological methods, offering a broad range of benefits [7]. The concentration of AgNPs during corn growth significantly influenced their uptake and accumulation, with increasing concentrations from 20 to 60 ppm resulting in enhanced shoot and root lengths, expanded leaf surface area, and higher chlorophyll content [8]. In addition to their growth-promoting effects, AgNPs have long been recognized as highly effective antibacterial agent against a wide range of microorganisms [9]. Both AgNPs and Ag^+^ ions exert antimicrobial activity by damaging microbial RNA and DNA, thereby inhibiting replication. Owing to their strong antibacterial properties, AgNPs are effective in controlling various infections and have also shown considerable potential in managing fungal diseases by inhibiting fungal growth and reproduction through disruption of essential cellular functions [10]. Their nano scale size facilitates improved penetration and interaction with fungal cells, enabling effective control of infections caused by *Fusarium verticillioides* [11]. Moreover, their ability to target specific biological processes in fungi without adversely affecting plant cells, combined with their broad-spectrum antimicrobial activity, highlights AgNPs as a promising strategy for the management of fungal infections [10].

AgNPs have shown a range of shapes, including rods, wires, spheres, and cubes and typically range in sizes from 3 to 100 nm. They can be readily synthesized through the thermal reduction of silver salts, such as 2-ethylhexyl carbamic acid, in which Ag^+^ ions are directly reduced to metallic Ag^0^ [12]. However, microwave heating has been reported to be more efficient than conventional thermal heating due to its lower energy consumption, improved operational efficiency, reduced cost, more precise temperature control, faster processing, and thermal uniformity [13]. Previous studies have shown that soil application of silica nanoparticles (SiO_2_-NPs) at rates up to 10 g kg^− 1^ significantly enhanced corn growth and yield [14], while the residual effects of various nanomaterials have been suggested as eco-friendly strategies to improve degraded soil quality and increase corn productivity [15]. Moreover, the incorporation of recycled waste in nanoparticle form has been reported to improve soil properties, enhance soil productivity and immobilize heavy metals. In parallel, effective nitrogen (N) management plays a critical role in improving N utilization efficiency (NUE) and achieving high grain yields in corn [16, 17], with higher rates of mineral N fertilizer being associated with improved yield components [18] and reduced incidence of certain fungal diseases [19, 20]. Therefore, the combined application of AgNPs and mineral N fertilizer represents an innovative approach to improve nutrient uptake, strengthen disease resistance, and further improve corn growth and productivity beyond the effects of N fertilizer alone. This study aimed to optimize crop yield while minimizing ear rot disease incidence through the appropriate mineral N application rates and AgNPs treatments.

## Materials and methods

### Study area

An experimental trial was carried out at the Ismailia Agricultural Research Station (30°35’30” N, 32°14’50” E; 10 m a.s.1.), Agricultural Research Center (ARC), Egypt, during the summer seasons of 2022 and 2023. Climatic data for the experimental site during the two seasons were collected from the NASA POWER database (https://power.larc.nasa.gov/data-access-viewer/) and are presented in Table 1. Prior to sowing, surface soil sample (0–30 cm) was collected to determine the physical and chemical properties of the soil, as summarized in Tables 2 and 3. These samples were collected at the same time and analyzed using methods of Black [21] by the Water and Soil Research Institute, ARC. Soil texture was determined by using the textural triangle based on the proportions of gravel, sand, silt and clay. The previous winter crop was wheat in both seasons.

**Table 1:** Solar radiation, maximum and minimum temperature, wind speed and evapotranspiration at the Ismailia Agricultural Research Station, ARC, 2022 and 2023 summer seasons

Month	2022					2023				
	SR	TX	TN	WS	ET0	SR	TX	TN	WS	ET0
June	30.0	35.1	20.6	3.0	8.2	30.0	35.8	19.6	3.0	8.5
July	29.0	37.8	23.5	2.8	8.5	29.0	38.4	22.6	2.8	8.8
August	26.8	36.7	23.8	2.7	7.8	26.8	37.2	22.8	2.7	8.0
September	23.4	33.9	21.3	2.8	6.6	23.4	34.3	19.9	2.9	6.8
October	18.9	29.2	18.1	2.7	4.8	18.9	29.3	16.7	2.6	4.9

*SR* Solar radiation (MJ/m^2^/day), *TX and TN* Maximum and minimum temperature, respectively (^0^ C), *WS* Wind speed (m/s), *ET0* Evapotranspiration (mm/day)

**Table 2:** Soil physical properties of the experimental sites of 2022 and 2023 summer seasons

Soil analysis	2022	2023
Coarse gravel %	72.40	71.40
Fine sand %	20.50	21.60
Silt %	3.30	3.20
Clay %	3.80	3.80
Texture	Sandy	Sandy

**Table 3:** Soil chemical properties of the experimental sites of 2022 and 2023 summer seasons

Soil analysis	2022	2023
Organic matter %	0.45	0.51
pH	7.75	7.82
E.C. ds/m	0.92	1.01
Soluble cations (mmolic/l)		
Ca^+ 2^	4.20	4.33
Mg^+ 2^	1.77	1.82
Na^+^	2.36	2.50
K^+^	0.93	1.02
Soluble anions (mmolic/l)		
HCO_3_^−^	2.11	2.25
Cl-	2.60	2.70
SO_4_^−2^	4.60	4.88
Available macro nutrients (ppm)		
N	17.21	20.23
P	4.82	5.66
K	62.44	72.25

### Microcrystalline cellulose and its uses

Microcrystalline cellulose (MCC) was purchased from WINLAB Laboratory Chemical Co. (UK), silver nitrate (AgNO_3_) was obtained from Aldrich Co. (Germany), and sodium hydroxide (NaOH) pellets were sourced from Sigma-Aldrich (Germany). All other chemicals, solvents, and materials were analytical grade and used as received without further purification. AgNPs was synthesized by microwave irradiation of an aqueous solution of silver alkyl carbamate (2.0 wt %) for 130 s at a power of 90 W [13]. This is definitely modified from another protocol [22]. AgNPs synthesis, solution A was prepared by dissolving 0.75 g of AgNO_3_ in 100 ml of deionized water. Solution B was prepared by dissolving 1 g of MCC in 100 ml of deionized water containing 0.2 g of NaOH per 1 g of MCC, with stirring at 75 °C for 5 min. Subsequently, solution A was added drop wise to solution B and kept at the same temperature under stirring for an additional 15 min. A visible color change from colorless to pale yellow and finally to dark brown, indicating the successful synthesis of AgNPs. The resulting solution was stored in the refrigerator for further analyses. During microwave-assisted reduction, Ag^+^ ions in the silver alkyl carbamate complex were converted to metallic Ag^0^ nanoparticles, accompanied by the decomposition of the complex and the release of carbon dioxide and 2-ethyl-1-hexylamine, as shown in Eq. (1).

1 $$\:2\left({C}_{8}{H}_{17}NHOCO\right)\:Ag+2{H}_{2}O)\to\:{4C}_{8}{H}_{17}{NH}_{2}+2Ag+2{CO}_{2}+{O}_{2}$$

### Characterization of AgNPs

The absorbance of AgNPs was analyzed using ultraviolet-visible spectroscopy (UV-Vis) spectroscopy (TG 80, Germany). The synthesized AgNPs exhibited a strong absorption peak, attributed to their excitation compared to the blank sample of MCC. The particle shape and size distribution of MCC-stabilized AgNPs were analyzed using transmission electron microscopy (TEM; JEOL-JEM-1230; Japan). A high-throughput solution of AgNPs was subjected to centrifugation at 20,000 rpm for 60 min to obtain AgNPs in powder form. The resulting AgNPs powder was examined using scanning electron microscopy (SEM; Quanta 400, Oxford, UK) to study the morphological traits of AgNPs. Elemental analysis of the AgNPs powder was conducted using energy-dispersive X-ray (EDX) with the SEM instrument. The average hydrodynamic particle size of AgNPs was determined using a zetasizer instrument (Nano-Sizer SZ90, Malvern Instrument Ltd., UK). Zeta potential determination of AgNPs was performed using dynamic light scattering (DLS) to provide information on the stabilization or aggregation of the MCC-coated AgNPs, ensuring their stability and preventing aggregation.

#### Analytical characterization

The morphological properties and elemental compositions of AgNPs were analyzed using a field-emission scanning electron microscope (FEG-SEM; Quanta FEG-250, Czech Republic)equipped with an EDX analyzer (TEAM model). TEM analysis (JEOL-1230, Japan) was performed to confirm nanoparticle morphology and size distribution. The crystalline structure of AgNPs was investigated using X-ray diffractometer (XRD; Germany).

### Experimental design and management

The experiments consisted of a combination of three mineral N fertilizer rates (240, 264, and 288 kg N ha^− 1^) and 4 AgNPs foliar spray treatments (control without AgNPs¸ 25, 50, and 75 ppm of AgNPs). Because it clearly shows the concentration of a material in a solution—one part of the solute per million parts of the entire solution by weight or volume—the unit “ppm” (parts per million) is regarded as common and extensively used. In chemistry and environmental sciences, it is most frequently used to express extremely diluted concentrations, such as those of nutrients, contaminants, or nanoparticles. In contrast to the unusual and perplexing unit “cm/l,” adopting “ppm” in scientific processes assures clarity and reproducibility while avoiding ambiguity. For clear communication, “cm/l” must be substituted with “ppm” or another common concentration unit like “mg/L” or “ml/L”. A randomized complete blocks design with split plot arrangement and three replications was used. Mineral N fertilizer rates were assigned to the main plots, while AgNPs were allocated to subplots. Each subplot comprised of six rows (0.70 m wide and 3.0 m long), resulting in a plot area of 12.6 m².

The yellow corn hybrid T.W.C. 352 was used as test material. Sowing was performed on June 5^th^ and June 7^th^ for the 2022 and 2023 seasons, respectively, with two kernels planted per hill at a depth of 2–5 cm and 20 cm spacing between hills. Plants were thinned to one per hill before the first irrigation, achieving a plant density of 48,000 plants ha^− 1^, which is recommended under Egyptian conditions. Drip irrigation was used throughout the growing periods.

Mineral N fertilizer was applied as ammonium nitrate (33.5% N) in two equal splits, before the initial and subsequent irrigations. The recommended N rate (288 kg N ha^− 1^) served as the high-input treatment, while the reduced rates (240 and 264 kg N ha^− 1^) were included to evaluate their effects on corn yield and ear rot disease incidence. Monocalcium superphosphate (15.5% P_2_O_5_) and potassium sulfate (48% K_2_O) were applied at a rate of 144 kg ha^− 1^ before sowing during field preparation. AgNPs were foliar sprayed three times at 15, 30, and 45 days after sowing. All other agronomic practices were carried out according to the recommendations of the Egyptian Ministry of Agriculture and Land Reclamation. Corn plants were harvested on September 25th and 28th in the 2022 and 2023 seasons, respectively.

### Related traits measurements

Plant sampling was conducted following the procedures described in *Statistical Procedures for Agricultural Research* [23]. Random sampling within experimental plots ensured representative and reliable data collection.

#### Phenological traits and ear leaf area

Days to 50% tasseling were recorded as the number of days from sowing until 50% of plants in each subplot showed visible tassels. Days to 50% silking were recorded as the number of days from sowing until 50% of the plants in each subplot reached the silking stage. Ear leaf area (dm^2^) was determined 75 days after sowing using the formula: leaf length × leaf width × 0.75, based on measurement from five plants per subplot [23, 24].

#### Grain yield and yield components

At harvest, ten randomly selected ears from the four inner rows of each subplot were used to determine ear weight (g), grain weight per ear (g), 100-grain weight (g), and shelling percentage (%).

$$\:Shelling\:\left(\%\right)=\frac{Grain\:Weight}{Ear\:Weight}\:\times\:100$$

Grain yield (t ha^− 1^) was determined based on total grain weight per subplot and extrapolated to a hectare basis.

#### Ear rot disease

Five ears per subplot were assessed for ear rot infection. Infection percentage and disease severity were calculated following standard formulas given by Canellas et al. [25].

$$\:Infection\:Percentage\left(\%\right)=\frac{Number\:of\:infested\:ears}{Total\:ears}\:\times\:100$$

$$\:Disease\:Percentage\left(\%\right)=\frac{Average\:number\:of\:infested\:grain\:in\:ear}{Average\:total\:grain\:in\:ear}\:\times\:100$$

Disease severity was categorized using a 1–7 scale given by Reid et al. [26] as follows,

1 = 0%, 2 = 3%, 3 = 4 to 10%, 4 = 11 to 25%, 5 = 26 to 50%, 6 = 51 to 75%, and 7 = more than 75% of grains affected.

### Statistical analysis

Although the specified experimental strategy—a randomized complete block design (RCBD) with a split-plot layout and three replications—is useful for organizing the experiment and managing variability. The approach guarantees appropriate replication and randomization, which are crucial for lowering bias and boosting result reliability. As a result, As a result, the design facilitates reliable data collection and interpretation. A combined analysis across two growing seasons was conducted after testing the homogeneity of error mean squares. As the year × treatment interaction was not significant, data were pooled across seasons. Analysis of variance (ANOVA) was performed using MSTAT software (version 8.0.1), and mixed models were applied to account for random effects [27]. Treatment means were compared using the least significant difference (LSD) test at the 5% probability level [23].

## Results and discussion

### Structural and elemental analysis

**Fig. 1:**
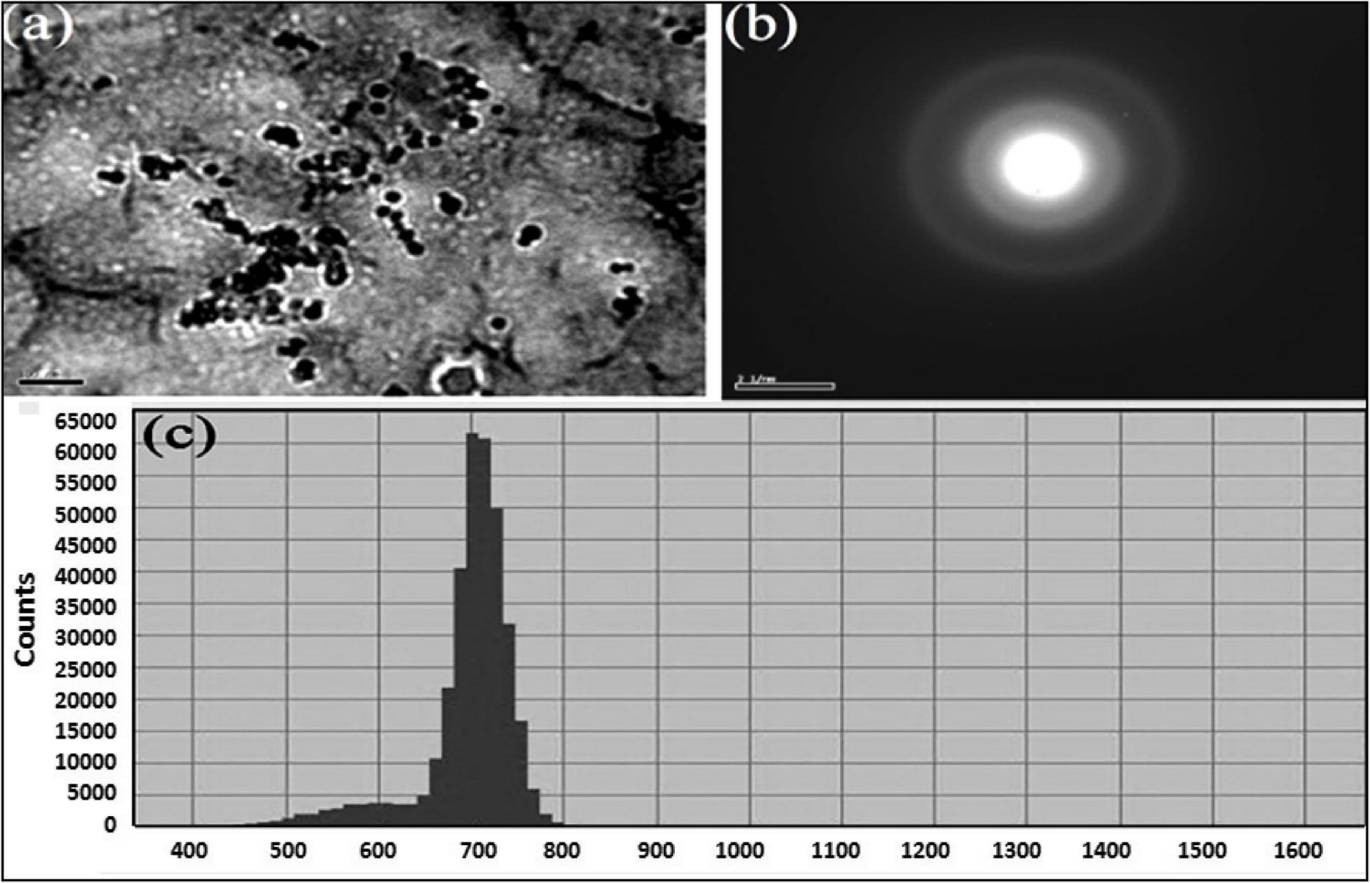
TEM analysis (**a**), selected area electron diffraction (**b**), and particle size distribution (**c**) of AgNPs

The TEM examination revealed that AgNPs ranged in size from 12 to 21 nm (Fig. 1). Figure 1a indicated that the harvested plant contained very low quantity of silver. The XRD pattern in Fig. 1b showed four distinct peaks at 2θ = 38.29°, 44.24°, 64.46°, and 77.17°, indicating a highly crystalline, face-centered cubic form of AgNPs. The diffraction peaks corresponded to (1,1,1), (2,0,0), (2,2,0), and (3,1,1) planes of Ag^0^, confirming the successful synthesis and integration of AgNPs into plant tissue.

**Fig. 2:**
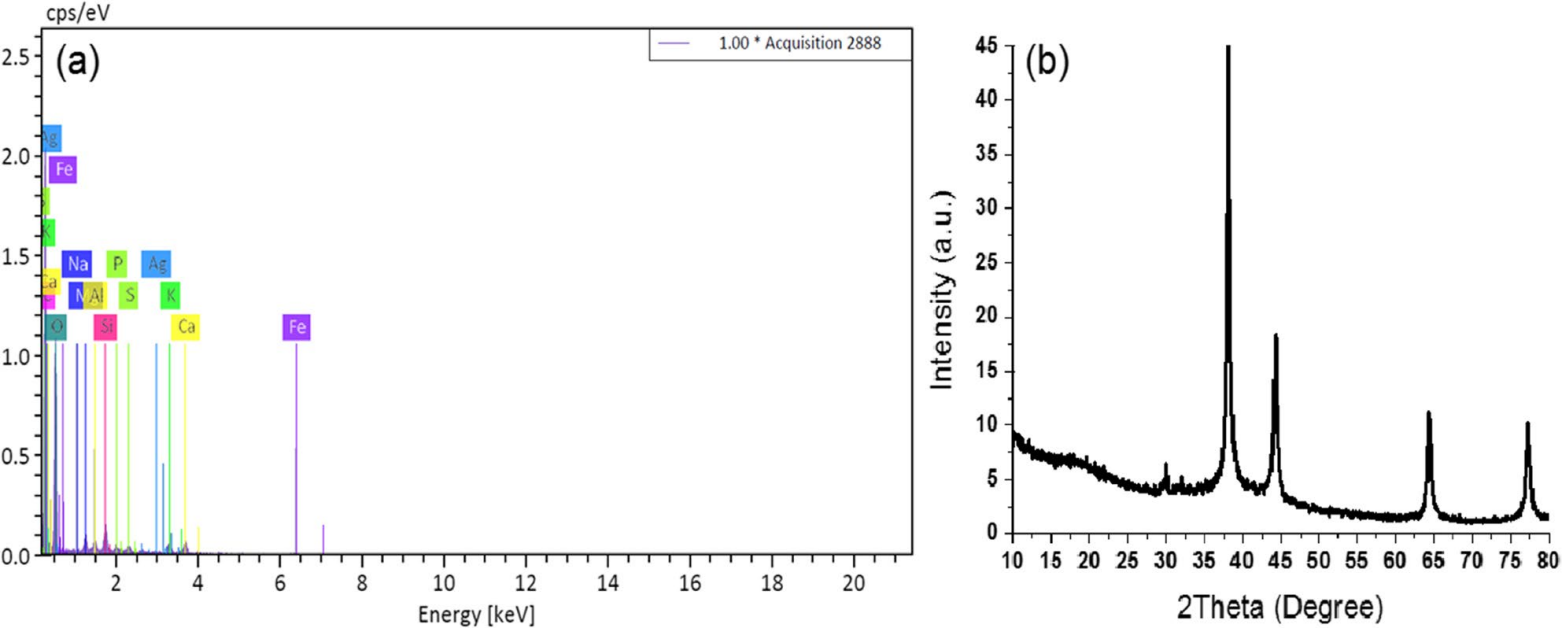
EDX analysis of plant tissue loaded with AgNPs (**a**) and XRD pattern of AgNPs (**b**)

The EDX analysis of the scanned AgNPs sample (Fig. 2) showed no visible peaks for residual silver, indicating that plant tissues absorbed the investigated AgNPs up to a concentration of 75 ppm in irrigation water without leaving detectable residues. This suggests complete uptake and integration of AgNPs at the tested concentrations. The characterization results validate the formation of AgNPs with a specific crystalline structure within plant tissue, holding promise for future applications in nanotechnology and biomedicine.

Further confirmation of the presence of AgNPs in plant tissue was obtained through SEM analysis, which revealed the distribution and morphology of the nanoparticles within the tissue matrix. The crystallite diameter of AgNPs was calculated using Scherrer’s formula [14], showing an increase in diameter as the concentration of silver alkyl carbamate increased from 0.5 to 6%. Understanding the absorption and distribution of AgNPs in plant tissues offers valuable insights for agricultural practices and environmental safety assessments.

Corn plants may respond differently to higher concentrations of AgNPs in irrigation water. Long-term studies on the effects of AgNPs on corn plants could provide valuable insights into their impact on crop growth and interactions with plant-associated pathogens [28]. Controlled experiments with varying concentrations of AgNPs can help researchers understand how these nanoparticles interact with plant tissues over time. The potential antimicrobial properties of these AgNPs could be explored for various biomedical and agricultural applications.

### Phenological traits and ear leaf area

#### Effects of mineral N fertilizer rates

**Fig. 3:**
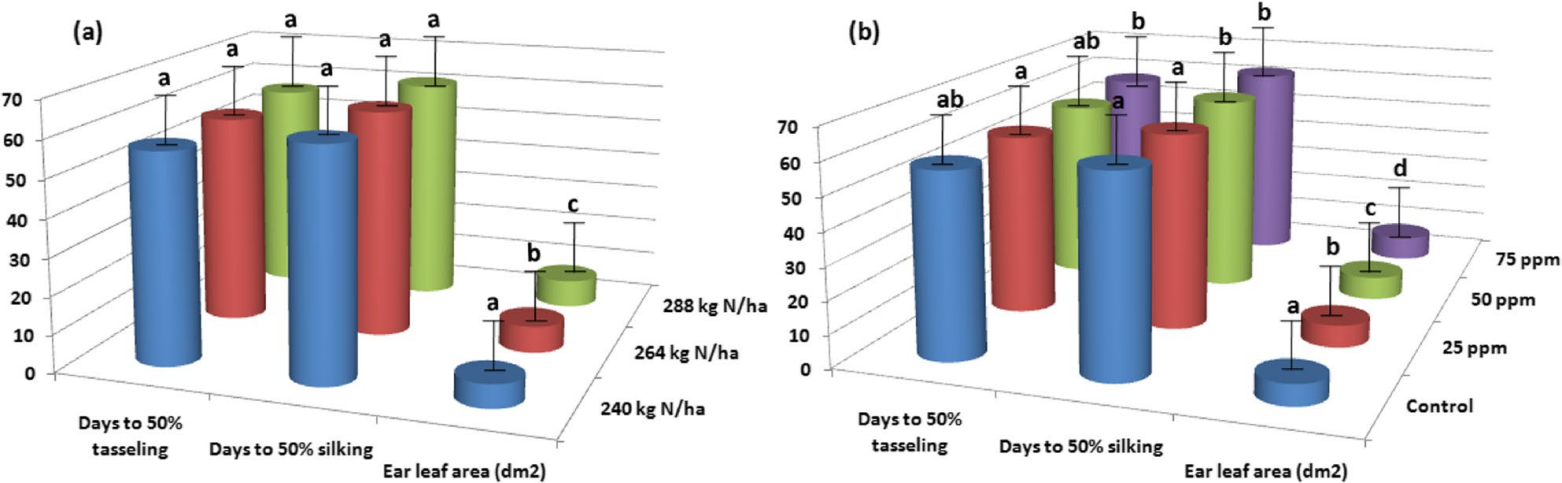
Days to 50% tasseling and 50% silking, and ear leaf area as affected by N fertilizer rates (**a**), LSD 5% for days to 50% tasseling is ns, LSD 5% for days to 50% silking is ns, and LSD 5% for ear leaf area is 0.33, and days to 50% tasseling and 50% silking, and ear leaf area as affected by AgNPs rates (**b**), LSD 5% for days to 50% tasseling is 1.10, LSD 5% for days to 50% silking is 0.88, and LSD 5% for ear leaf area is 0.20

Mineral N fertilizer rates significantly affected ear leaf area in the combined data across both growing seasons; however, days to 50% tasseling and 50% silking showed no significant response (Fig. 3a). Ear leaf area increased by 19.55% with the application of 288 kg N ha^− 1^ compared to lower rate (240 kg N ha^− 1^).These findings suggest that mineral N fertilizer rates play a crucial role in enhancing ear leaf area in corn plants, indicating that proper fertilization can improve vegetative growth and productivity. The timing of tasseling and silking was unaffected by the different mineral N fertilizer rates, suggesting that other environmental or genetic factors may regulate these reproductive development processes.

The importance of N as a fertilizer in influencing physiological and biochemical processes that impact plant growth and development is well documented [29, 30], and our results align with these previous findings. The lack of response in tasseling and silking timing suggests that these phenological events are more strongly regulated by photoperiod and temperature than by nitrogen availability within the range tested. Any delay in tasseling could impact the reproductive phase of corn plants, potentially affecting pollination synchrony and ultimately grain yield. Similarly, delayed silking may affect the physiological processes of corn plants, prolonging the reproductive stage and potentially impacting overall crop development and yield potential.

#### Effects of AgNPs rates

The application of different rates of AgNPs significantly influenced days to 50% tasseling and 50% silking, as well as ear leaf area in the combined data across both seasons (Fig. 3b). Days to 50% tasseling were 55.84 days with 25 ppm of AgNPs and 57.11 days with 75 ppm of AgNPs, representing a 2.27% increase that was not statistically significant. These results suggest that increasing AgNPs rates within this range does not substantially alter the timing of tassel emergence in corn. This relatively modest and statistically non-significant impact of higher AgNPs concentrations on tasseling is likely due to limited impacts on the physiological process governing tassel development. The application of 25 ppm AgNPs had no discernible impact on silking timing compared to the control treatment, indicating that this concentration does not substantially affect the developmental stage of silking in corn. In contrast, the application of 50 or 75 ppm AgNPs significantly delayed silking compared to 25 ppm or the control treatment. This delay may result from increased physiological stimulation or stress responses induced by higher AgNPs concentrations, which can alter plant developmental processes.

Regarding ear leaf area, there was a 13.60% increase with the application of 75 ppm AgNPs compared to the control treatment. This increase could be attributed to the beneficial effects AgNPs at 75 ppm on improving nutrient uptake efficiency, promoting cell division and expansion, and reducing pathogen stress, all of which contribute to enhanced leaf growth and larger ear leaf area. Previous studies have demonstrated AgNPs’ ability to enhance plant growth and development by promoting nutrient uptake and increasing photosynthetic activity [31, 32]. Additionally, the antimicrobial properties of AgNPs may have contributed to the improved ear leaf area by reducing disease pressure on the plants [33]. Overall, these findings suggest that AgNPs can be a valuable tool in agriculture for enhancing crop productivity, particularly in stressful environments such as sandy soils.

#### Interaction between mineral N fertilizer rates and AgNPs rates

The combined data from both seasons indicated that the interaction between mineral N fertilizer rates and AgNP rates did not significantly impact days to 50% tasseling or 50% silking in corn (Table 4). However, this interaction had a significant effect on ear leaf area. The combination of highest mineral N fertilizer rate with 75 ppm AgNPs resulted in the largest ear leaf area compared to all other treatment combinations. The synergistic effect may arise from the complementary mechanism of high mineral N fertilizer, which provides plenty of nutrients for vegetative growth, and 75 ppm of AgNPs, which improve nutrient uptake efficiency, stimulate physiological processes, and reduce disease or environmental stress. The combined effects of high mineral N fertilizer and AgNPs enhance physiological processes such as photosynthesis and leaf expansion, leading to an increased ear leaf area. This increased leaf area enhances the plant’s ability to absorb light and generate energy, promoting better growth and development.

**Table 4:** Effect of the interaction between mineral N fertilizer and AgNPs rates on days to 50% tasseling and 50% silking, and ear leaf area, combined data across the two seasons

Mineral *N* fertilizer	AgNPs	Days to 50% tasseling	Days to 50% silking	Ear leaf area (dm^2^)
240 kg N/ha	Control	56.00	60.33	5.70
	25 ppm	55.66	60.31	6.20
	50 ppm	56.00	61.33	6.45
	75 ppm	57.00	62.22	6.60
264 kg N/ha	Control	56.22	60.33	6.80
	25 ppm	55.66	60.32	6.90
	50 ppm	56.10	61.33	7.20
	75 ppm	57.12	62.22	7.50
288 kg N/ha	Control	57.10	60.44	6.90
	25 ppm	56.20	60.45	7.20
	50 ppm	56.30	61.22	7.80
	75 ppm	57.22	62.01	7.95
LSD 5%		ns	ns	0.31

Furthermore, the antimicrobial properties of AgNPs may reduce pathogen burden, particularly under challenging sandy soil conditions, enabling the plant to allocate more resources toward leaf growth rather than defense mechanisms. The synergistic use of AgNPs with different mineral N fertilizer rates could potentially alter corn ear morphology and size, with implications for final grain yield. Future research should investigate whether these vegetative growth improvements translate into enhanced reproductive development and grain production under various environmental conditions.

### Grain yield and yield components

#### Effect of mineral n fertilizer rates

**Fig. 4:**
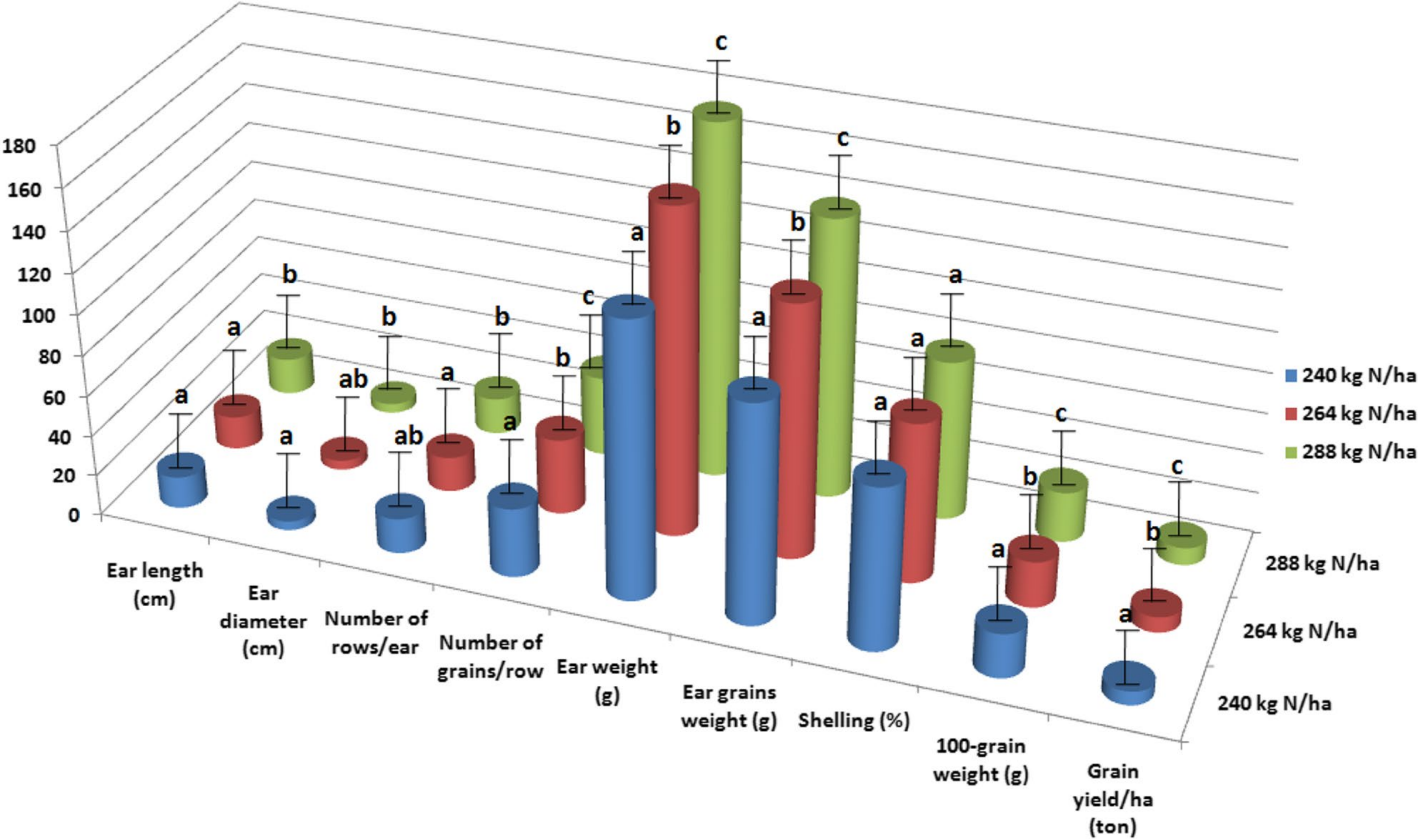
Grain yield and yield components as affected by mineral N fertilizer rates. LSD 5% for ear length is 1.20, LSD 5% for ear diameter is 0.35, LSD 5% for number of rows/ear is 0.74, LSD 5% for number of grains/row is 2.01, LSD 5% for ear weight is 10.80, LSD 5% for ear grains weight is 6.02, LSD 5% for shelling is ns, LSD 5% for 100-grain weight is 1.01, and LSD 5% for grain yield/ha is 0.80

Mineral N fertilizer rates had a significant impact on grain yield and yield components of corn, except for shelling percentage, in the combined data across both growing seasons (Fig. 4). No significant differences were observed between the 240 and 264 kg N ha^− 1^ fertilizer rates for ear length, ear diameter, and number of rows per ear. However, the highest N rate (288 kg N ha^− 1^) significantly increased grain yield and yield components. These results indicate that while ear morphological traits such as length, diameter, and number of rows per ear reached a plateau between 240 and 264 kg N ha^− 1^, the higher N rate of 288 kg N ha^− 1^ further enhanced grain filling and other physiological processes, leading to increased grain yield and improved yield components beyond those achieved at lower rates. Compared to the application of 240 kg N ha^− 1^, the 288 kg N ha^− 1^ treatment resulted in notable increases in ear length (15.05%), ear diameter (8.20%), number of rows per ear (4.09%), number of grains per row(15.75%), ear weight (28.98%), ear grains weight (28.62%), 100-grain weight(14.56%), and grain yield per hectare(30.08%). Higher mineral N rates enhance essential physiological processes in corn plants, such as photosynthesis and metabolite accumulation, leading to improved ear characteristics and ultimately higher yields [34]. The 100-grain weight, a genetically regulated trait showed significant variation in response to different mineral N rates. This variation affects the source-sink relationship, particularly when N supply is insufficient [35]. The 100-grain weight plays a crucial role in the kernel development and the final kernel size during the grain-filling period [36].Gene expression regulates this trait, and adequate N availability appears to optimize the genetic potential for grain weight.

#### Effect of AgNPs rates

**Fig. 5:**
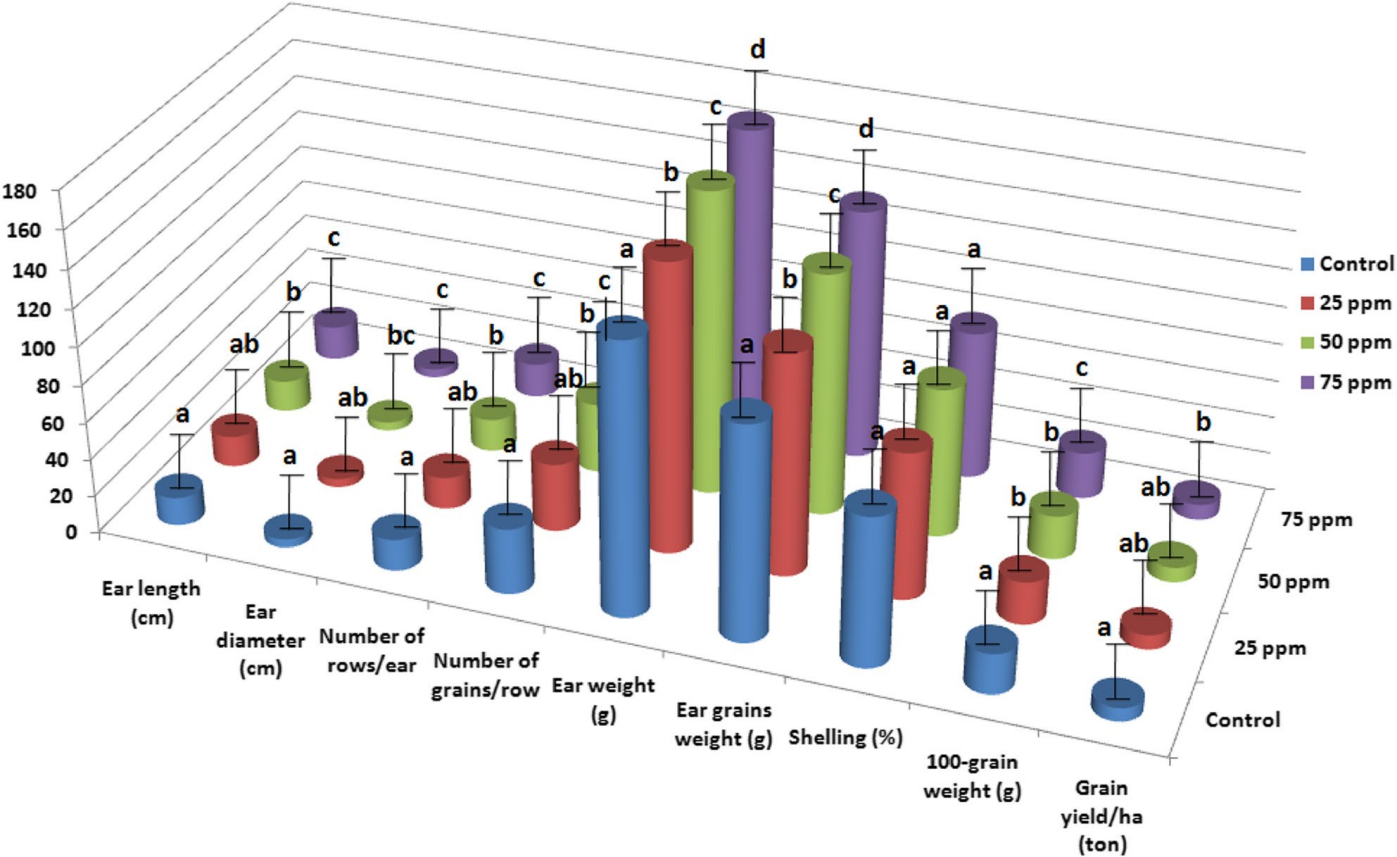
Grain yield and yield components as affected by AgNPs rates. LSD 5% for ear length is 0.98, LSD 5% for ear diameter is 0.19, LSD 5% for number of rows/ear is 0.75, LSD 5% for number of grains/row is 1.50, LSD 5% for ear weight is 5.80, LSD 5% for ear grains weight is 5.50, LSD 5% for shelling is ns, LSD 5% for 100-grain weight is 1.18, and LSD 5% for grain yield/ha is 0.97

AgNPs significantly influenced grain yield and yield components of corn, except for shelling percentage, in the combined data across both seasons (Fig. 5). Application of 75 ppm of AgNPs resulted in notable improvements in grain yield and yield components compared to the control treatment. The low concentration of 25 ppm of AgNPs was insufficient to produce measurable impact on ear traits and grain yield, possibly because the dosage did not significantly enhance nutrient uptake, physiological processes, or disease resistance compared to the control treatment. No significant differences were observed between the control treatment and 25 ppm of AgNPs for ear length, ear diameter, number of rows per ear, number of grains per row, and grain yield per hectare. In contrast, the high rate of 75 ppm AgNPs increased ear length, ear diameter, number of rows per ear, number of grains per row, ear weight, ear grain weight, 100-grain weight, and grain yield per hectare by 23.52%, 6.01%, 10.34%, 11.14%, 16.31%, 18.00%, 16.04%, and 16.38%, respectively, compared to the control treatment. The higher concentration of 75 ppm of AgNPs greatly increased nutrient uptake, stimulated plant physiological processes, and provided stronger antibacterial effects, all of which improved growth traits and yield components. Higher rates of AgNPs promoted superior growth and development in corn plants, suggesting that plants may exhibit more robust development when exposed to higher rates of AgNPs.

The beneficial effects of AgNPs may operate through both direct and indirect mechanisms. Higher rates of AgNPs may have indirect benefits on soil fertility, structure, and bacterial abundance [37], as well as direct effects on cell wall, membrane, cytoplasm, photosynthesis, respiration levels, protein synthesis, and plant development through enhanced nutrient uptake and production [7]. The application of AgNPs increased the 100-grain weight of corn, a genetic trait regulated by gene expression, indicating that AgNPs can positively influence corn crop yield potential. The enhanced 100-grain weight may result from AgNPs’ ability to improve nutrient absorption and stimulate plant growth, resulting in larger and heavier kernels [38]. Furthermore, AgNPs may directly influence the physiological mechanisms involved in kernel development and grain filling. In contrast, the control treatment yielded the lowest results for most traits in both seasons, likely due to the absence of the antimicrobial protection provided by AgNPs, potentially leading to increased pathogen pressure and reduced plant growth and productivity.

#### The interaction between mineral N fertilizer rates and AgNPs rates

The combined data from both seasons showed a significant impact of the interaction between mineral N fertilizer rates and AgNPs rates on grain yield and yield components, except for number of rows per ear, number of grains per row, and shelling percentage (Table 5). The highest values for ear weight, ear grains weight, 100-grain weight, and grain yield per hectare were observed with combination of 75 ppm AgNPs and 288 kg N ha^− 1^, followed 75 ppm AgNPs and 264 kg N ha^− 1^. The synergistic effect of high concentration of AgNPs (75 ppm) combined with higher N fertilizer rates (288 and 264 kg N ha^− 1^) improved nutrient uptake efficiency, improved plant physiological functions, and reduced disease stress, resulting in higher ear weight, grain weight, and overall grain yield. This interaction significantly influenced ear weight, shelling percentage, 100-grain weight, and grain yield per hectare. Higher rates of both N and AgNPs had positive effect on ear weight, ear grain weight, 100-grain weight, and grain yield per hectare, indicating that the combined application of AgNPs and increased N rates improve the processes of plant growth and development.

**Table 5:** Effect of the interaction between mineral N fertilizer and AgNPs rates on grain yield and yield components, combined data across the two seasons

Mineral N fertilizer	AgNPs	Ear length (cm)	Ear diameter (cm)	Number of rows/ear	Number of grains/row	Ear weight (g)
240 kg N/ha	Control	14.10	4.33	17.12	31.15	112.90
	25 ppm	15.33	4.25	17.13	34.33	135.12
	50 ppm	16.12	4.45	17.25	35.55	144.17
	75 ppm	17.40	4.55	17.86	35.83	151.11
264 kg N/ha	Control	15.00	4.55	16.11	36.15	153.25
	25 ppm	16.33	4.66	17.22	37.22	156.38
	50 ppm	16.44	4.75	17.34	37.38	162.41
	75 ppm	18.00	4.84	18.58	39.45	171.52
288 kg N/ha	Control	16.18	4.60	17.23	38.24	166.19
	25 ppm	17.31	4.71	17.38	38.11	170.17
	50 ppm	18.44	4.82	18.33	40.06	180.24
	75 ppm	20.52	4.89	19.25	42.01	184.16
LSD 5%		2.21	0.50	ns	ns	7.3

Mineral N fertilizer		AgNPs	Ear grains weight (g)	Shelling (%)	100-grain weight (g)	Grain yield/ha (ton)
240 kg N/ha		Control	89.11	79.93	20.33	5.64
		25 ppm	106.15	78.56	21.32	6.73
		50 ppm	116.18	80.58	22.00	7.25
		75 ppm	120.21	79.55	23.15	7.24
264 kg N/ha		Control	122.15	79.71	21.33	7.28
		25 ppm	116.20	78.05	22.33	7.68
		50 ppm	128.22	78.95	23.15	7.97
		75 ppm	135.33	78.90	24.16	8.33
288 kg N/ha		Control	130.20	78.34	22.66	8.33
		25 ppm	133.25	78.30	24.33	8.42
		50 ppm	144.35	80.09	25.14	8.84
		75 ppm	147.41	80.04	27.33	9.16
LSD 5%			9.0	ns	1.62	1.32

The antimicrobial properties of AgNPs reduce disease stress, enabling plants to allocate more resources to yield components such as ear weight, grain weight, and grain yield per hectare. Simultaneously, improved nutrient availability from increased N fertilization supports better cell division, expansion, and grain filling. AgNPs and high mineral N fertilizer work synergistically to improve plant physiological processes, reduce disease pressure, and increase nutrient absorption and assimilation. Through enhanced root uptake, metabolic stimulation, and antimicrobial defense against pathogens, this combination promotes more efficient nitrogen utilization, resulting in superior growth and yields. This translates to increased N use efficiency and successful yield enhancement. Previous research has shown that corn productivity can be improved by combining NPK fertilizers with AgNPs [39]. When AgNPs and NPK are combined, fertilizers become more readily absorbed and more effective than conventional biochemical fertilizers [40], suggesting a promising avenue for integrated nutrient management strategies in corn production systems.

Although AgNPs positively impact crop development and disease control, their accumulation in soil and effects on soil microbiota were underexplored in this study. This was due to a focus on immediate agricultural benefits and insufficient monitoring of silver residues and microbial community changes over time. The authors acknowledge the importance of these environmental factors for sustainability and safety, and assert that future research should include accurate measurements of silver levels in grains, soil, and discharge, as well as an assessment of nanoparticles’ interactions with soil, mobility, transformation, bioavailability, and associated health risks. It is also crucial to evaluate the long-term impacts on microbial communities and soil health to address environmental and food safety concerns in agricultural practices.

### Ear rot disease severity

#### Effect of mineral N fertilizer rates

**Fig. 6:**
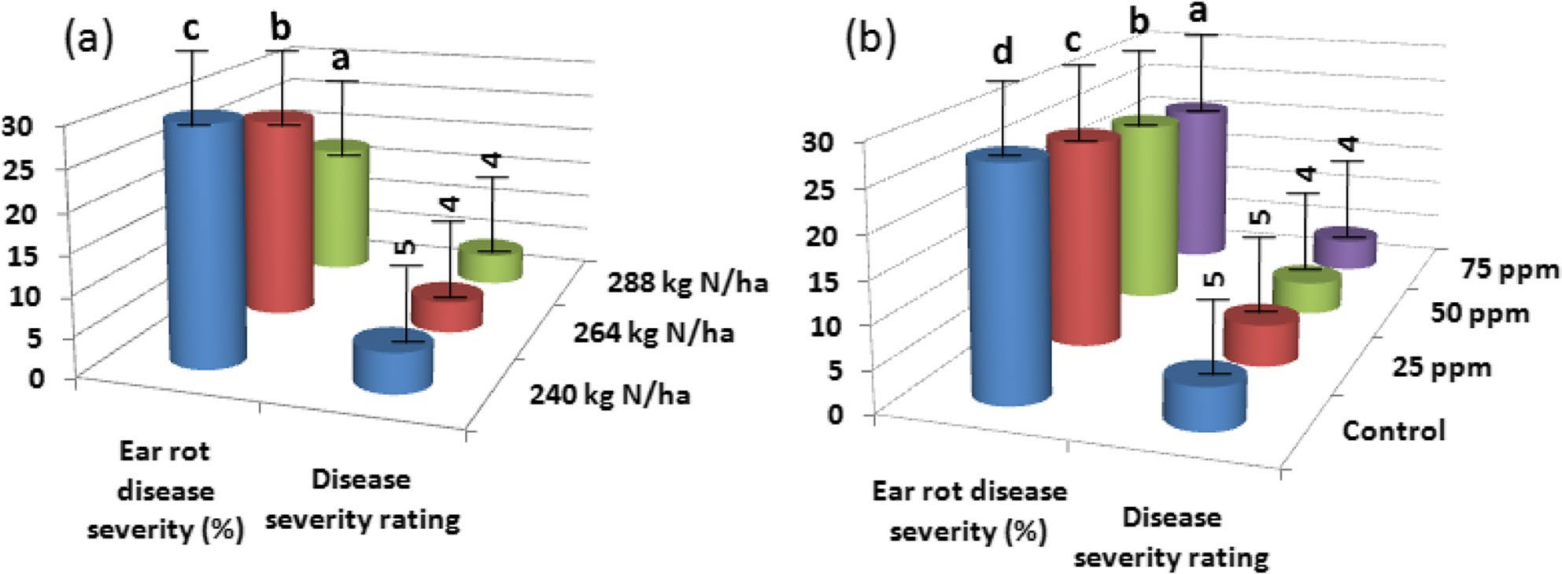
Ear rot disease severity and disease severity rating as affected by mineral N fertilizer rates (**a**), LSD 5% for ear rot disease severity is 1.30, and ear rot disease severity and disease severity rating as affected by AgNPs rates (**b**), LSD 5% for ear rot disease severity is 1.21. Disease severity was measured built on the subsequent graded rule as follows: 1 = 0, 2 = 1 near to 3%, 3 = 4 near to 11%, 4 = 11 near to 25%, 5 = 26 near to 50%, 6 = 51 near to 75%, 7 = more than 75% disease contagion (Reid et al. 2001)

The combined data across both growing seasons showed that mineral N fertilizer rates significantly affected the severity of corn ear rot disease (Fig. 6a). Increasing mineral N fertilizer rates from 240 to 288 kg N ha^− 1^ resulted in a significant 41.28% reduction in ear rot disease severity. The disease severity rating decreased from 5 at 240 kg N ha^− 1^ to 4 at 288 kg N ha^− 1^, indicating improved disease resistance at higher N rates. Higher mineral N rates promote stronger plant health and enhanced disease resistance, leading to reduced ear rot disease severity [41]. The reduction in disease severity rating could be attributed to several mechanisms. First, adequate nitrogen nutrition may enhance plant defense mechanisms through a dilution effect, where increased nutrient availability strengthens plant resistance to pathogens. Second, higher mineral N rates may result in more vigorous and healthier plants overall, which are better equipped to resist pathogen invasion and colonization. Third, increased mineral N rates may enhance nutrient uptake and utilization efficiency by corn plants, improving overall plant vigor and stress tolerance, thereby further reducing disease susceptibility [42].

#### Effect of AgNPs rates

The combined data across both growing seasons demonstrated that AgNPs application rates significantly affected corn ear rot disease severity (Fig. 6b). Foliar application of AgNPs at 75 ppm significantly reduced ear rot disease severity by 23.30% compared to the control treatment. The disease severity rating decreased from 5 in the control treatment to 4 when AgNPs were applied at 75 ppm. The reduction in ear rot disease severity can be attributed to the well-documented antimicrobial properties of AgNPs, which inhibit the growth and development of pathogens responsible for the disease. The decrease in disease severity rating results from the toxic effects of AgNPs on the fungal pathogens causing ear rot disease, which impair their growth and reproduction [43]. AgNPs exhibit high capacity to generate reactive oxygen species (ROS), which damage pathogen DNA, disrupt cell membranes, and inhibit protein synthesis [44].

Moreover, AgNPs application increases the production of phenolic compounds such as phenylpropanoids in plant tissues, which reduce plant infections by enhancing plant resistance mechanisms and modifying antioxidant systems [45]. These phenolic compounds and associated enzymes have direct antimicrobial effects against plant pathogens [46]. The combined effect of direct antimicrobial activity and induced plant resistance contributes to the observed reduction in ear rot disease severity.

#### Interaction between mineral n fertilizer rates and AgNPs rates

The severity of corn ear rot disease was significantly influenced by the interaction between mineral N fertilizer rates and AgNPs application rates in the combined data across both growing seasons (Table 6). The highest disease severity was observed in the control treatment (no AgNPs) combined with the lowest N fertilization rate (240 kg N ha^− 1^). Conversely, the lowest disease severity was observed with the combination of 75 ppm AgNPs and 288 kg N ha^− 1^. The disease severity rating decreased from 5 in the control treatment with 240 kg N ha^− 1^ to 4 with the combined application of 75 ppm AgNPs and 288 kg N ha^− 1^.

**Table 6:** Effect of the interaction between mineral N fertilizer and AgNPs rates on ear rot disease severity (%) and disease severity rating, combined data across the two seasons

Mineral *N* fertilizer	Ag NPs	Ear rot disease severity (%)	Disease severity rating
240 kg N/ha	Control	32.49	5
	25 ppm	31.16	5
	50 ppm	28.49	5
	75 ppm	27.41	5
264 kg N/ha	Control	26.82	5
	25 ppm	25.13	5
	50 ppm	24.02	4
	75 ppm	22.70	4
288 kg N/ha	Control	22.33	4
	25 ppm	19.01	4
	50 ppm	16.16	4
	75 ppm	12.49	4
LSD 5%		3.37	---

Disease severity was measured built on the subsequent graded rule as follows: 1=0, 2=1 near to 3%, 3= 4 near to 11%, 4= 11 near to 25%, 5= 26 near to 50%, 6= 51 near to 75%, 7= more than 75% disease contagion (Reid et al. 2001)

This synergistic effect likely results from the complementary mechanisms of enhanced nutrient uptake through increased mineral N rates and the antimicrobial properties of AgNPs. Higher N fertilization improves overall plant health and resistance by providing adequate nutrients for defense compound synthesis and stress response mechanisms. Simultaneously, the antimicrobial properties of AgNPs directly inhibit the growth and activity of the pathogens causing ear rot disease. These components work synergistically to strengthen plant defense systems and reduce disease severity.

The enhanced disease resistance from adequate nitrogen nutrition creates a more robust host defense system, while AgNPs provide direct pathogen suppression through their antimicrobial mechanisms. This dual approach—strengthening host resistance while simultaneously suppressing pathogen activity—proves more effective than either strategy alone. The results suggest that utilizing AgNPs in conjunction with higher mineral N rates can effectively combat ear rot disease by simultaneously enhancing plant defenses and directly suppressing pathogen populations. This integrated approach may offer a sustainable and innovative solution for managing fungal diseases in corn production systems, reducing reliance on conventional fungicides while maintaining or improving disease control efficacy.

## Conclusion

In sandy soil conditions, vertical intensification can help reduce ear rot disease and boost corn grain production by combining mineral N fertilizer with a AgNPs spray. The application of high rates of both mineral N and AgNPs led to significant growth and yield improvements, while also reducing ear rot disease incidence. Although the combination of AgNPs with high mineral nitrogen fertilizer increases resistance to ear rot diseases in maize cultivated on nutrient-poor sandy soil, this study was limited in that it did not assess the Product Health Index. Evaluating this index would give more understanding of how this combination impacts the general health of plants. AgNPs offer a practical and effective solution for enhancing crop health and yield in sandy soil, as they can be easily applied as a foliar spray.

## Data Availability

The data that support the findings of this study are available from the corresponding author upon reasonable request.
